# ApaltAI: a web-based diagnostic system with a sequential voting architecture for detecting anthracnose and scab in avocado fruit

**DOI:** 10.3389/fpls.2026.1736123

**Published:** 2026-02-24

**Authors:** Mikjael Moreano, Angel Sosa, David Mauricio, Luis Rivera, José Santisteban

**Affiliations:** 1 Faculty of Engineering, Universidad Peruana de Ciencias Aplicadas (UPC), Lima, Peru; 2 Department of Computer Science, Universidad Nacional Mayor de San Marcos, Lima, Peru; 3 Mathematical Sciences Laboratory, Universidade Estadual do Norte Fluminense, Rio de Janeiro, Brazil

**Keywords:** avocado, convolutional neural networks, deep learning, disease detection, image processing

## Abstract

Avocado (*Persea americana* Mill.), with a global production estimated at 10.4 million tons in 2023, suffers annual losses of 20-30% due to diseases such as anthracnose (*Colletotrichum gloeosporioides*) and scab (*Sphaceloma perseae*), resulting in substantial economic impacts for major producing countries (Mexico, Peru, and Colombia). This study introduces an advanced system that integrates a binary sequential voting architecture (VotingBS) with a fully functional web application, for the automated identification of two high-incidence diseases: anthracnose and scab, both of which critically affect fruit quality and yield. The proposed VotingBS architecture implements a hierarchical two-stage classification strategy. In the first stage, a five-model deep learning ensemble differentiates between healthy and diseased fruits. In the second stage, another ensemble determines which of the two diseases is present. For this purpose, a collection of 674 labeled fruit images was used for training and validation. Experimental results demonstrate outstanding model performance, achieving key metrics such as 98.92% precision, 98.89% recall, and 99.03% accuracy, significantly outperforming traditional approaches. Moreover, the solution was deployed through a web app featuring dedicated modules for crop management, phytosanitary analysis, and disease diagnosis. This architecture enhances the system’s practical utility and facilitates its adoption by farmers, field technicians, and agricultural monitoring agencies. Overall, this work demonstrates how combining hybrid deep learning models with accessible digital platforms can revolutionize plant disease diagnostics, fostering a more efficient, automated, and resilient precision agriculture.

## 1 Introduction

Avocado (*Persea Americana* Mill.) is a widely consumed fruit, particularly across the Americas, and is highly valued for its bioactive properties and health benefits. Its pulp is rich in monounsaturated and polyunsaturated fatty acids, phytosterols, and fat-soluble vitamins, compounds that have been shown to positively influence metabolic health and contribute to the prevention of chronic diseases (Ahmed et al., 2025). In 2023, avocado ranked as the second most exported tropical fruit worldwide, with a volume of 2.8 million tons, surpassed only by pineapple at 3.2 million tons. Mexico and Peru remain the leading exporters (FAO, 2024).

Furthermore, avocado is highly susceptible to infection by various pathogenic fungi, both in the field and during postharvest stages, leading to substantial losses in fruit yield and quality (Silva et al., 2025). One of the most prevalent diseases affecting this crop is anthracnose (*Colletotrichum gloeosporioides*), caused by fungi of the *Colletotrichum* genus, which can infect fruit tissues, leading to rot (Colín-Chavez et al., 2024). Another significant disease is scab (*Sphaceloma perseae*), which affects both fruit and leaves in warm and humid climates, diminishing crop quality and yield (Chellappan, 2024).

Conventionally, the diagnosis of these pathologies has relied on visual inspection by agronomists, a process that is subjective, time-consuming, and difficult to scale, particularly for smallholder farmers who often lack immediate access to specialist expertise. This diagnostic bottleneck delays timely intervention, exacerbating yield and quality losses (Demilie, 2024). While precision agriculture and Deep Learning (DL) offer promising alternatives, their translation into practical, accessible tools for specific crops like avocado remains limited. There is a pronounced gap between the development of accurate DL models in controlled research settings and their deployment as usable, reliable diagnostic aids in real-world agricultural scenarios. This work addresses this gap by developing ApaltAI, an integrated system that combines a novel, high-accuracy decision architecture with a functional web application designed specifically for end-users in the avocado production chain.

Artificial intelligence is increasingly shaping agriculture, with applications that range from optimizing irrigation through machine learning (Villagomez et al., 2024) to the automated identification of plant diseases. In this field, convolutional neural networks (CNNs) have demonstrated high efficacy in image analysis, proving useful not only in agriculture but also in domains such as medicine. In clinical practice, for instance, CNNs have achieved performance levels comparable to human specialists in detecting ocular pathologies (Moreno-Lozano et al., 2024) and brain abnormalities (Rodríguez et al., 2024). Their key advantage lies in the ability to automatically extract and learn visual patterns, making them particularly well suited to agricultural problems where diseased crops often exhibit wide morphological variability.

The strong performance of CNNs has encouraged extensive research on applying deep learning (DL) to crop disease detection. Sultan et al. (2025) developed LeafDNet, a model based on Xception architecture, trained with 5,491 images of crops such as rose, mango and tomato. Their system achieved 99% precision and 98% accuracy. Huang et al. (2023) developed a hybrid model for disease detection in tomato plants; the proposed model, FC-SNDPN, reached a precision of 97.59%. Moussafir et al. (2022) designed a model to identify diseases in tomatoes using a hybrid architecture and a dataset of 14,526 images. After evaluating seven architectures, the two best-performing models were combined, resulting in 98.1% precision. Butt et al. (2025) developed a system for detecting diseases in citrus fruits; the hybrid model combining DenseNet201 and a C-SVM (Support Vector Machine) classifier yielded the highest accuracy on their fruit dataset, achieving 99.2%. Finally, Banjar et al. (2025) introduced the E-AppleNet model for disease detection in apple crops, using 3,168 images from the PlantVillage dataset. Utilizing the EfficientNetV2 architecture, their system achieved a 99% accuracy rate.

While DL has shown promise for avocado disease detection (e.g., Campos-Ferreira and González-Camacho, 2021), existing studies often focus solely on model accuracy, leaving a critical void: the integration of robust detection models into accessible, end-to-end platforms ready for field use. Furthermore, many approaches employ single, complex classifiers that must simultaneously distinguish between healthy tissue and multiple diseases, a task prone to error propagation. To overcome these limitations, this study introduces ApaltAI, a comprehensive web-based diagnostic system. The core of ApaltAI is the VotingBS (Binary Sequential Voting) architecture, a novel decision model designed to enhance reliability by decomposing the diagnosis into a hierarchical, two-stage process. This design is inherently more robust and is operationalized through a purpose-built web application, making the advanced diagnostic capability directly accessible to farmers and technicians. Therefore, the central theme of this article is the development and validation of an integrated, accessible system (ApaltAI) for avocado disease detection, whose performance and practicality are driven by its innovative VotingBS decision engine.

The combination of CNNs with sophisticated techniques has given rise to hybrid systems, which now represent a promising approach for classifying agricultural images and detecting plant diseases. Recent studies on disease detection in potato and apple cultivation have shown that hybrid and models outperform approaches relying solely on CNNs, achieving significant improvements in both accuracy and efficiency (Tiwari et al., 2020; Bansal et al., 2021).

To bridge the identified gap between accurate model development and field-deployable solutions, this work pursues two interconnected objectives: (1) to design and validate the VotingBS (Binary Sequential Voting) architecture, a novel hybrid decision framework that enhances diagnostic reliability by decomposing the classification into a hierarchical, two-stage process; and (2) to engineer and deploy ApaltAI, a fully functional web-based diagnostic system built around this architecture, making the technology accessible to end-users. Consequently, the primary contributions of this work are threefold: (a) the VotingBS architecture, a robust decision system that strategically combines multiple deep learning models with a sequential, weighted voting logic to mitigate error propagation; (b) the ApaltAI integrated system, a deployable software platform featuring a modular three-tier design and specialized modules that translate the VotingBS model into a practical diagnostic service; and (c) a comprehensive experimental benchmark, demonstrating that the integrated system not only outperforms state-of-the-art singular and hybrid models but also establishes a new performance benchmark (precision, recall, accuracy >98.9%) for avocado fruit disease detection.

This article is divided into six sections. Section two reviews the background and related works. Section three describes the materials and methods, including the design of the VotingBS architecture, the web application and the validation process. Section four reports the experimental results, followed by section five, which discusses these findings in the context of related work and outlines the system’s contributions. Finally, section six summarizes the main conclusions and suggests directions for future research.

## 2 Background and related works

**Figure 1:**
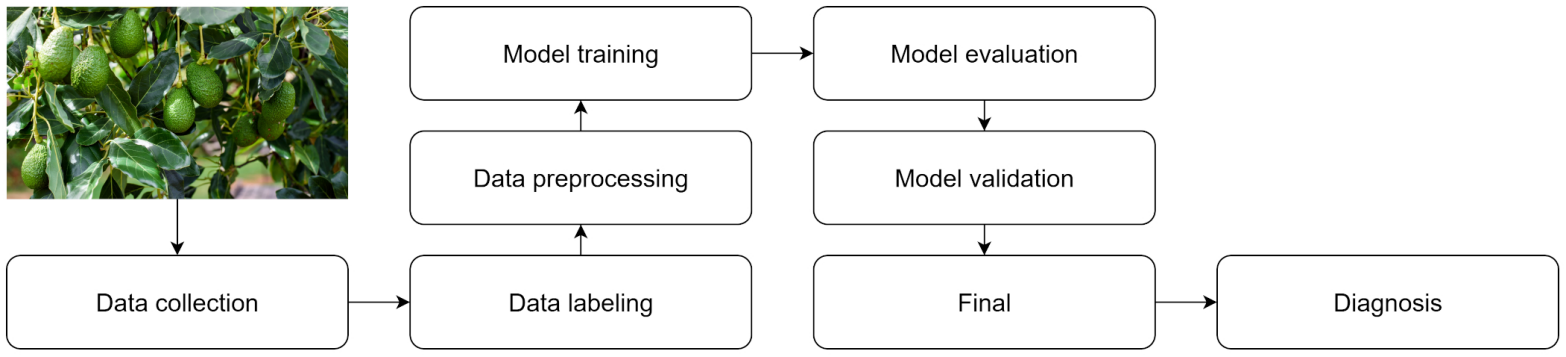
General process for crop disease detection.

The application of CNNs for plant disease identification involves a carefully structured sequence of stages, each of which plays a critical role in achieving reliable diagnostic performance. In general, the studies analyzed on this topic follow the process sequence shown in **Figure 1**. Analyzing this common workflow across studies is crucial for identifying both established best practices and persisting limitations, thereby framing the specific research gap addressed by our proposed system.

The process flow followed consists of:

- *Data collection*: The process begins with acquiring images of affected crops. These images are either captured directly in the field using cameras and mobile devices or obtained from open-access repositories such as Kaggle and PlantVillage. For example, Banjar et al. (2025) utilized 3,168 images from PlantVillage, categorized into four distinct disease classes.
- *Data labeling*: Each image is accurately annotated to identify the specific disease it presents. This detailed annotation enables the model to learn and differentiate distinctive patterns for each disease. For instance, Saleem et al. (2022) performed detailed labeling of images from crops such as apple, avocado, grape, kiwi, and pear, creating the NZDLPlantDisease-v1 dataset, which comprises 20 different classes.
- *Data preprocessing*: Before initiating training, all images are subjected to a preprocessing pipeline that includes scaling, normalization, contrast adjustment, and/or noise removal, as highlighted in several studies (Alshammari et al., 2022; Kaya and Gürsoy, 2023). This step is crucial for improving image quality and ensuring the model is trained under optimal conditions, facilitating accurate identification of disease-related patterns. For example, Sholihati et al. (2020) applied data augmentation to enrich their dataset, resulting in a more robust system.
- *Model training*: Involves training the DL model with labeled, preprocessed images, enabling it to learn and differentiate the characteristic visual features of each disease.
- *Model evaluation and validation*: A dataset of unseen images serves to evaluate the trained model. This evaluation quantifies the accuracy and provides the basis for refining its architecture and hyperparameters to optimize performance.

The various related works have, in one way or another, followed the presented workflow. For training and validation purposes, these authors utilized datasets such as PlantVillage, which aggregates images of potatoes, tomatoes, apples, and strawberries, among other produce, across several classes; and own datasets containing various classes of images of potatoes, apples, olives, bananas, guavas, and mangoes, among others. A few studies also employed the New Plant Disease Dataset and the Potato Leaf Disease Dataset. In the quality analysis of potatoes, the VGG+LR model was formulated by Tiwari et al. (2020) using the PlantVillage dataset, and the VGG16 model was formulated by Sholihati et al. (2020) using their own dataset. The PlantVillage dataset was also used in the quality analysis of tomatoes with CNN-based models by Karthik et al. (2020); Agarwal et al. (2020), and in the DenseNet121 model proposed by Abbas et al. (2021). For apple care, own datasets were used in Hybrid models (DenseNet121, EfficientNetB7, EfficientNet) by Bansal et al. (2021), in the Xception + F-RCNN model by Khan et al. (2022), the MLP-CNN by Turkoglu et al. (2022), and the standard CNN by Vishnoi et al. (2023). Additionally, the PlantVillage dataset was used in the CNN model by Mahato et al. (2022), the DenseNet+1D-CNN model by Sai and Neeraja (2022), and the CNN + Unet model by Polly and Devi (2024). Other specific datasets were also used in apple analysis, such as the New Plant Disease Dataset in the AIE-ALDC model by Al-Wesabi et al. (2022), as well as a proprietary dataset by Banarase and Shirbahadurkar (2024) in MobileNetV2. Hari and Singh (2023) used a CNN-based model with their own dataset for the analysis of three types of fruits (Banana, Guava, Mango). We only observed one study concerning avocado quality classification: the MSCA-PSCO MobileNetV2 model developed by Mishra et al. (2022) using their own dataset. **Table 1** summarizes these 19 studies on DL-based disease detection in fruits using images and their performance results.

**Table 1:** DL studies for crop disease detection.

Study	Dataset	Crop type	Model	Results
Tiwari et al. (2020)	PlantVillage: 2,152 (3 classes)	Potato	VGG19+LR	Acc=97.8%
Karthik et al. (2020)	PlantVillage: 120,000 (4 classes)	Tomato	CNN	Acc=98%
Agarwal et al. (2020)	PlantVillage: 17,500 (10 classes)	Tomato	CNN	Acc=91.2%
Sholihati et al. (2020)	Own dataset: 5,100 (5 classes)	Potato	VGG16	Acc=91.31%
Abbas et al. (2021)	PlantVillage: 16,012 (10 classes)	Tomato	DenseNet121	Acc=97.11%
Bansal et al. (2021)	Own dataset: 3,642 (4 classes)	Apple	Hybrid (DenseNet121, EfficientNetB7, EfficientNet NoisyStudent)	Acc=96.25%
Mishra et al. (2022)	Own dataset: 19,460 (2 classes)	Avocado	MSCA-PSCO MobileNetV2	Acc=98.42%
Alshammari et al. (2022)	Own dataset: 3,400 (3 classes)	Olive	ViT+VGG16	Acc=97% Pre=98%
Al-Wesabi et al. (2022)	New Plant Disease Dataset: 9,714 (4 classes)	Apple	AIE-ALDC	Acc=99.20%
Khan et al. (2022)	Own dataset: 5,201 (10 classes)	Apple	Xception + F-RCNN	Acc=81.09%
Mahato et al. (2022)	PlantVillage: 32,950 (4 classes)	Apple	CNN	Pre=99,31%
Sai and Neeraja (2022)	PlantVillage: 8,875 (4 classes)	Apple; Grape; Potato; Strawberry	DenseNet +1D-CNN	Acc=97%
Turkoglu et al. (2022)	Own dataset: 1,192 (4 classes)	Apple	MLP-CNNs	Acc=99.2%
Hari and Singh (2023)	Own dataset: 1,791 (8 classes)	Banana; Guava; Mango	CNN	Acc=99.14%
Vishnoi et al. (2023)	Own dataset: 3,171 (4 classes)	Apple	CNN	Acc=98%
Mir et al. (2024)	Own dataset: 4,190 (8 classes)	Potato	CNN + RF	Acc=93.66%
Polly and Devi (2024)	PlantVillage: 8,631 (4 classes)	Tomato; Corn; Apple	CNN + UNet	Acc=98.01% Pre=99.5%
Banarase and Shirbahadurkar (2024)	Own dataset: 3,175 (4 classes)	Apple	MobileNetV2	Acc=99.36%
Sinamenye et al. (2025)	Potato Leaf Disease Dataset: 3,076 (7 classes)	Potato	EfficientNetV2B3 + ViT	Acc=85.06% Pre=82.86%

F-RCNN, Faster Region Convolutional Neural Network; Pre, Precision; DCNN, Deep Convolutional Neural Network; Acc, Accuracy; ViT, Vision Transformer.

The analysis in **Table 1** consolidates the remarkable progress of DL, particularly CNNs, in crop disease detection, with many models achieving accuracy rates above 95% across various crops. This establishes a strong technological precedent. However, three critical gaps relevant to our work can be observed: (1) a predominant focus on leaf diseases over fruit-specific pathologies; (2) a scarcity of studies dedicated to avocado, particularly targeting fruit diseases like anthracnose and scab; and (3) a strong emphasis on model accuracy in isolation, with fewer examples of complete, deployable systems tailored for end-user adoption. These gaps highlight the opportunity and necessity for the present study. Consequently, while leveraging the established efficacy of CNNs, our work introduces a novel sequential decision architecture (VotingBS) specifically designed to enhance robustness for fruit disease diagnosis and embeds it within a fully functional web application (ApaltAI). The adoption of deep learning-based diagnostic systems not only enhances disease identification accuracy but is also designed to lead to more sustainable crop management through accessible, timely diagnostics.

## 3 Materials and methods

### 3.1 Proposed detection architecture

An IT-based architecture is proposed for detecting scab and anthracnose in avocado fruits using image analysis. It employs deep learning-based image processing and follows five components: image acquisition, preprocessing, diagnosis module, the DL model and the diagnostic output.

**Figure 2:**
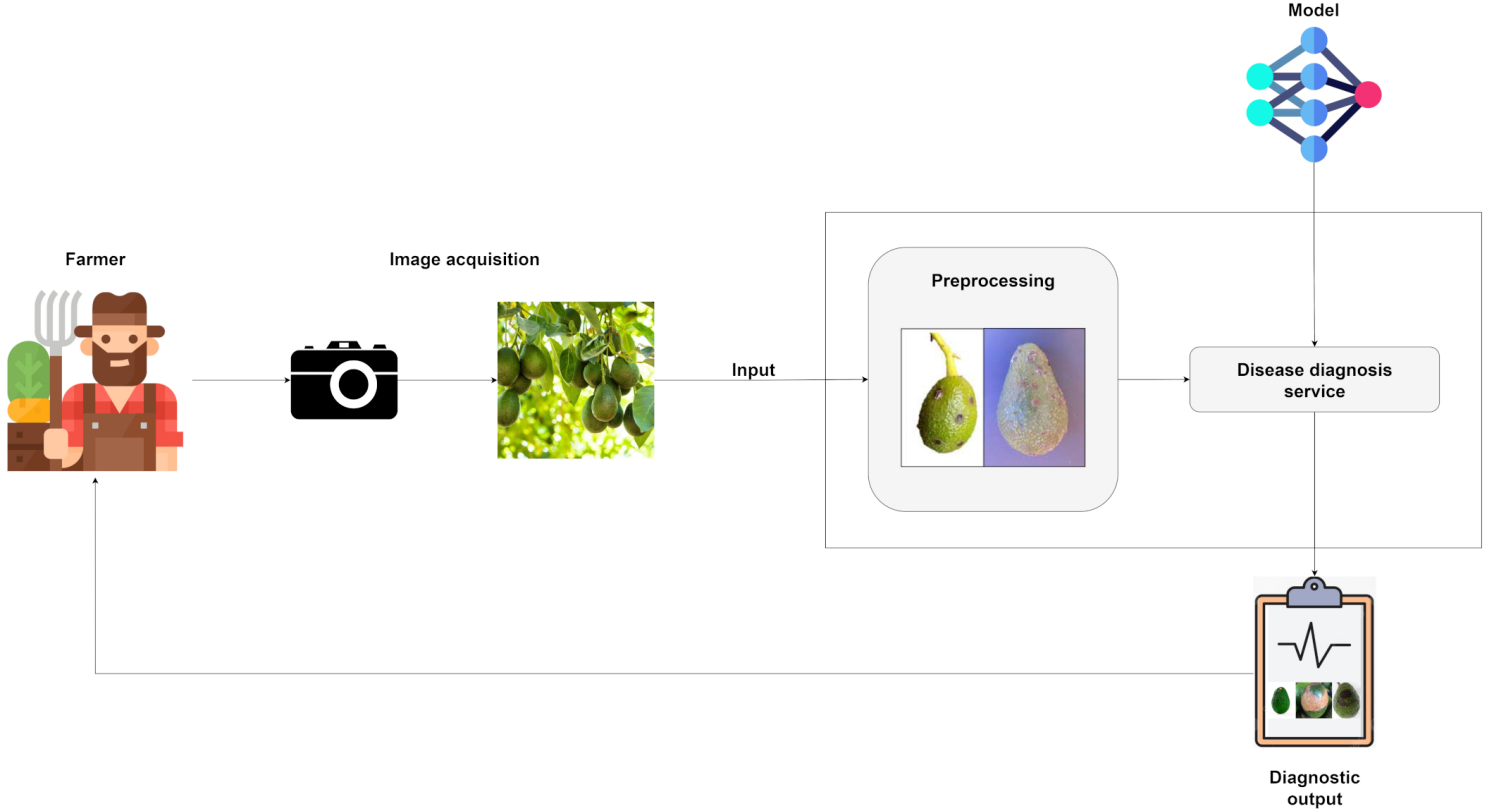
Proposed disease detection process.

**Figure 2** shows the workflow, starting with the farmer capturing fruit images, which are then preprocessed to enhance clarity and definition. The optimized data are analyzed by a diagnosis module powered by a pre-trained ensemble architecture that combines several DL models for classification. Based on this analysis, the system evaluates each image and determines the fruit’s condition, classifying it as healthy, affected by scab or affected by anthracnose. Finally, the diagnostic result is delivered to the farmer, enabling appropriate treatment decisions. The components of the system are described below (**Table 2**).

**Table 2:** Components of the detection process.

Component	Description
Image Acquisition	Digital cameras, drones and mobile devices now play a central role in agricultural monitoring, providing a practical means of capturing images in the field (Chen et al., 2021). Accurate diagnosis depends on the availability of high-quality images that clearly display critical indicators such as leaf spots, discoloration areas and irregular texture patterns (Rani et al., 2023).
Preprocessing	To facilitate analysis, a preprocessing pipeline is applied to highlight the most relevant features. The process begins with image resizing to match the input requirements of the pre-trained model architecture. This is followed by pixel value normalization, which standardizes data distribution and enhances training stability. Data augmentation is also incorporated through adjustments in lighting and contrast, helping reduce overfitting and improving the model’s adaptability to diverse visual conditions.
Model	Once preprocessed, the images are fed into a validated DL model. In this study, an ensemble architecture is used, integrating predictions from models such as DenseNet121, ResNet50, InceptionV3, VGG16, and EfficientNetB2. Model parameters are optimized during training using the Stochastic Gradient Descent (SGD) algorithm, which iteratively computes weight updates using random data subsets to minimize the loss function and promote effective convergence.
Disease diagnosis service	This service receives an input image and processes it using a DL model (either singular or hybrid) to generate a diagnosis of the disease.
Diagnostic output	Presents the diagnosis, including the detected disease, the model’s confidence level (expressed as a percentage or probability) and a history of previous diagnoses. Additionally, it provides information about the identified disease along with agronomic management recommendations and treatment options, all delivered through an interface.

The construction of the hybrid DL model VotingBS is carried out in two stages. First, each DL model is trained separately. Transfer learning is employed for this purpose, leveraging pre-trained weights to initialize the networks and fine-tune them for the specific task. In this study, five DL architectures are considered: DenseNet121, ResNet50, InceptionV3, VGG16, and EfficientNetB2. Second, a binary sequential voting architecture, called VotingBS, is constructed to analyze the input avocado image and generate a diagnosis of ‘Healthy’ or an outcome indicating affliction by scab or anthracnose. This architecture is described below.

### 3.2 VotingBS

**Figure 3:**
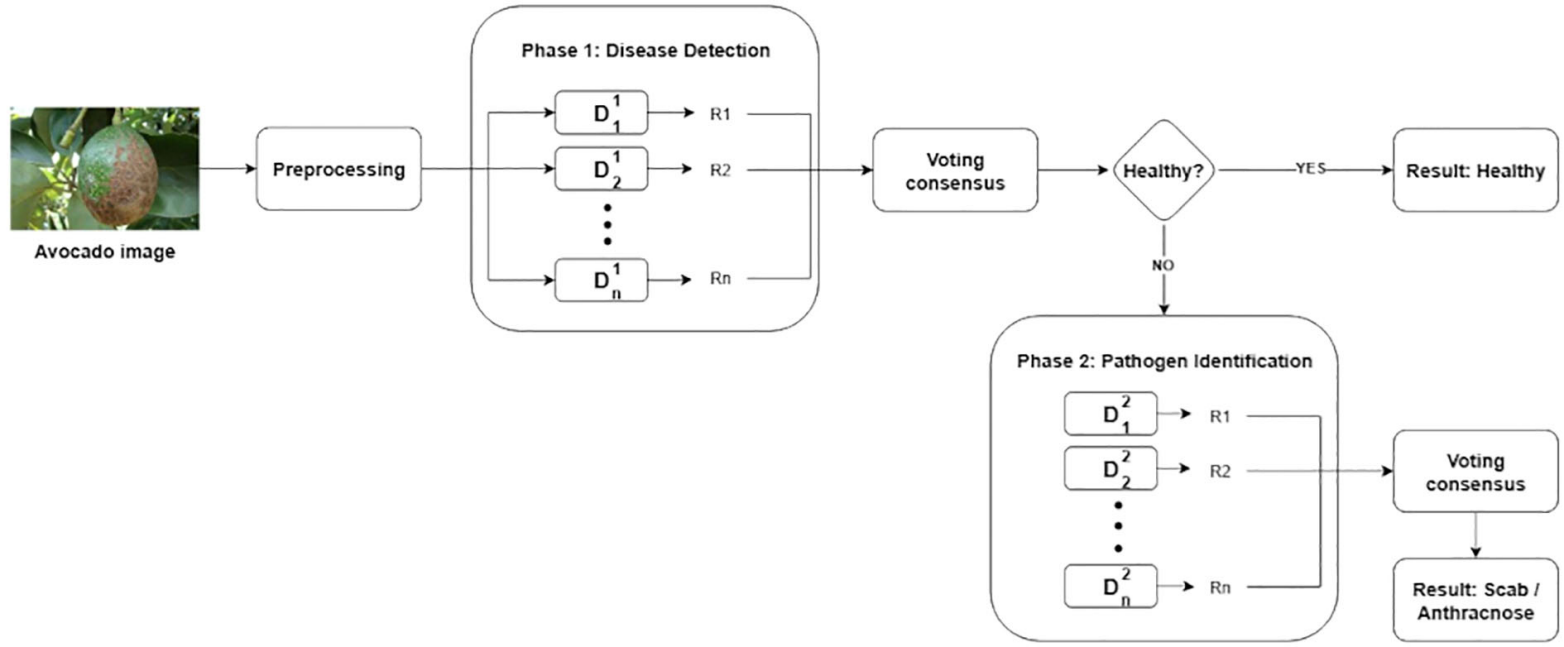
The VotingBS decision architecture.

This process leads to the construction of the VotingBS (Binary Sequential Voting) architecture. VotingBS is a hybrid decision system that orchestrates two sets of five binary DL models $(D_{i}^{1}$ and $D_{i}^{2},\;\;i=1,\ldots ,5)$ through a structured, two-phase voting scheme. The scheme operates as follows: In the first phase. A unanimous or majority decision of “healthy” concludes the process with that result. Otherwise, the system proceeds to the second phase. In this phase —where the avocado is considered unhealthy— the models $D_{i}^{2},\;\left(i=1,\;\ldots n\right)\;$ classify the image as either anthracnose or scab. Their outputs are again submitted to a voting process, which selects the majority decision as the final classification. This binary and sequential structure constitutes the VotingBS architecture and is illustrated in **Figure 3**. Therefore, VotingBS is not merely a *post-hoc* voting mechanism; it is an integral hybrid system where the specialized DL models and the sequential decision logic are co-designed. This justifies its direct comparison against singular DL models (which lack this decision structure) and other ensemble methods, as all represent distinct approaches to the classification task.

**Figure 3** illustrates the sequential two-phase voting process: a first voting stage to distinguish healthy from diseased fruit, and upon a diseased outcome, a second voting stage to discriminate between anthracnose and scab. In both voting phases, each DL model ( $D_{i},\;i=1,\ldots ,5)$ produces a classification result along with a confidence value, given by its individual precision ( $P_{i},\;i=1,\ldots ,5)$. These confidence values are normalized to obtain class-specific normalized weights (**Equation 1**).

(1) $$w_{i}=\frac{P_{i}}{\sum_{j=1}^{5}P_{j}\;\;}$$

Subsequently, the normalized weights are aggregated according to the predicted class, such as A and S. The final score for each class is then calculated as shown in Equations 2 and 3.

(2) $$Score\;A\;=\sum_{i=1}^{5}w_{i}\cdot I\left({\hat{y}}_{i}=A\right)\;$$

(3) $$Score\;S\;=\sum_{i=1}^{5}w_{i}\cdot I\left({\hat{y}}_{i}=S\right)$$

Where $I\left({\hat{y}}_{i}=k\right)$ is a function that equals 1 if ${\hat{y}}_{i}=k$ ( k=A or S) and 0 otherwise.

Finally, the class receiving the highest confidence score is assigned as the diagnostic result. This voting-based approach enables the integration of multiple model outputs and improves the overall classification accuracy by reducing the impact of erroneous predictions from any single model.

### 3.3 Web application

ApaltAI is a web-based application powered by CNNs, developed to identify pathologies in avocado fruits. The platform implements a classification scheme optimized to process images and generate diagnostic results. Its development addresses the need to provide farmers, particularly small-scale producers, with an accessible tool to identify plant pathologies, enabling the timely adoption of preventive or corrective measures with minimal latency.

#### 3.3.1 Logical architecture

**Figure 4:**
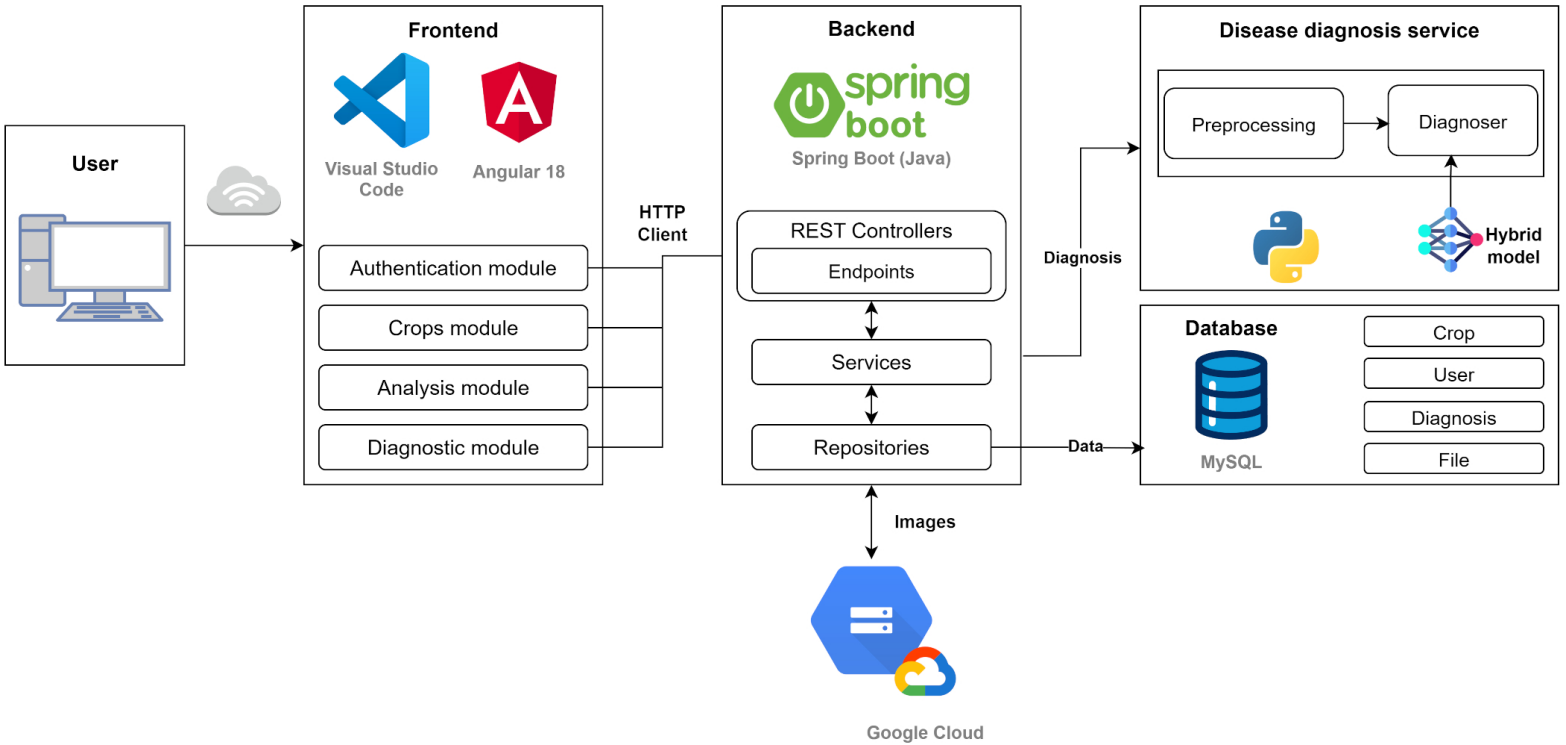
Web application architecture.

The architecture of ApaltAI follows a modular three-layer design (**Figure 4**), ensuring scalability, security, and efficiency in diagnostic processing:

- *Frontend layer*: A responsive web interface designed for non-technical users (e.g., farmers), optimized for both mobile and desktop devices. It allows intuitive image uploads and the visualization of diagnostic results.
- *Backend layer*: Implemented using Spring Boot (Java), this layer handles: business logic and workflow management; authentication via JWT (JSON Web Tokens); secure communication with other layers through RESTful APIs; and integration with storage services.
- *Diagnosis layer*: A specialized service developed with FastAPI (Python) that encapsulates the CNN-based classification model (TensorFlow/Keras). Key features include: (a) image preprocessing (normalization, data augmentation); (b) real-time inference using the trained model; and (c) generation of diagnostic outputs.

#### 3.3.2 Technologies used

The development of ApaltAI integrates four main components that collectively enable the full functionality of the application:

- Frontend: Developed using Angular 18 and the Bootstrap 5.3.2 styling framework, this component serves as the primary interaction point for farmers. It is designed to facilitate user interaction by allowing the upload of fruit images for analysis in JPEG and PNG formats.
- Backend: Built with Spring Boot 3.3.4, the backend forms the core of the application, managing business logic, user management, and session handling. Security is a priority, implemented via JWT to ensure secure communication between client and server and to restrict access to critical functions to authenticated users only. Additionally, RESTful APIs are used for managing crops, diagnostics, and related information.
- Diagnosis service: This specialized service is implemented using FastAPI 0.115.11 in Python and hosts the disease detection model developed with TensorFlow 2.19.0, supported by auxiliary libraries such as Scikit-learn 1.6.1 and Keras. The diagnosis service receives images from the backend, processes and analyzes each image to detect signs of disease and returns the results for user presentation.
- Data storage: The system uses MySQL for structured data storage, such as user records and diagnostic results, leveraging its scalability, high performance, and automated administration. For handling unstructured data (images), Google Cloud Storage is employed —a highly scalable solution that ensures fast and efficient access, even with growing data volumes. This dual-architecture approach optimizes both metadata processing and storage of critical visual resources for the model.

#### 3.3.3 Application modules

**Figure 5:**
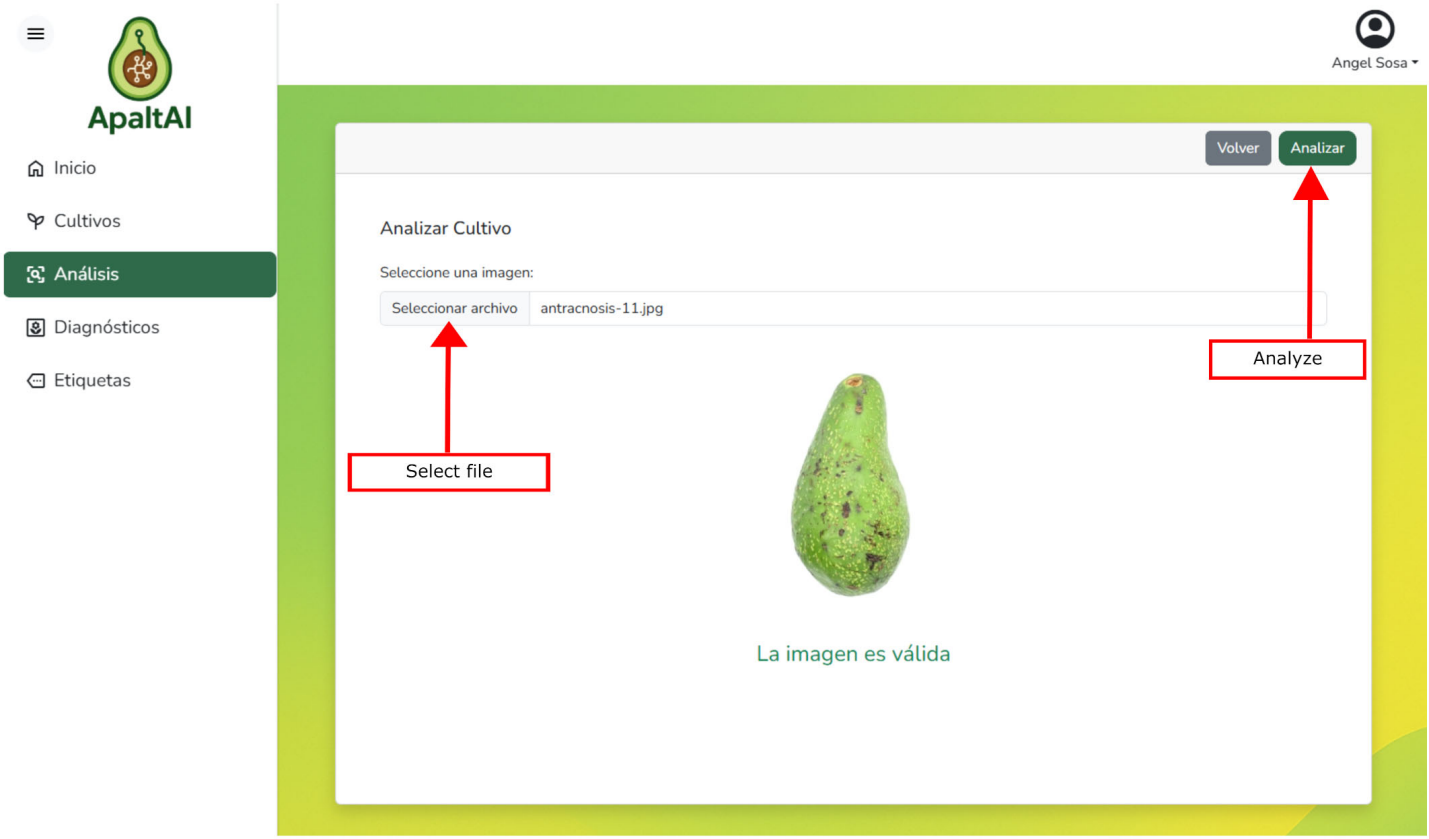
Interface of the analysis module.

The proposed web application integrates three main modules designed to facilitate user-system interaction for disease detection in avocado crops: (1) Crop Module, (2) Analysis Module, and (3) Diagnosis Module. Their key functionalities are described below:

- *Crop module*: This module allows users to register new crops and maintain ongoing monitoring through personalized notes. Each registered crop is displayed in a table, from which it can be consulted, edited, or deleted as needed. Over time, users can add annotations to record phenological events, environmental conditions, or other relevant occurrences. This annotation capability supports more organized crop management and helps maintain a useful historical record for future decision-making.
- *Analysis module*: Designed to process images in an automated and efficient manner. The process begins with the validation of the image uploaded by the user, checking for aspects such as format, minimum required resolution, and file size. This step ensures compliance with analytical requirements (see **Figure 5**). Once validated, the image proceeds through preprocessing to guarantee optimal input quality. The VotingBS architecture then analyzes the processed images to detect and classify their condition. The resulting data obtained can be saved in a relational database and subsequently forwarded to the diagnosis module for visualization and further analysis.

**Figure 5** illustrates the user interface for image upload and validation, a key component of the ApaltAI workflow that demonstrates the integration of the VotingBS architecture into a user-friendly process.

**Figure 6:**
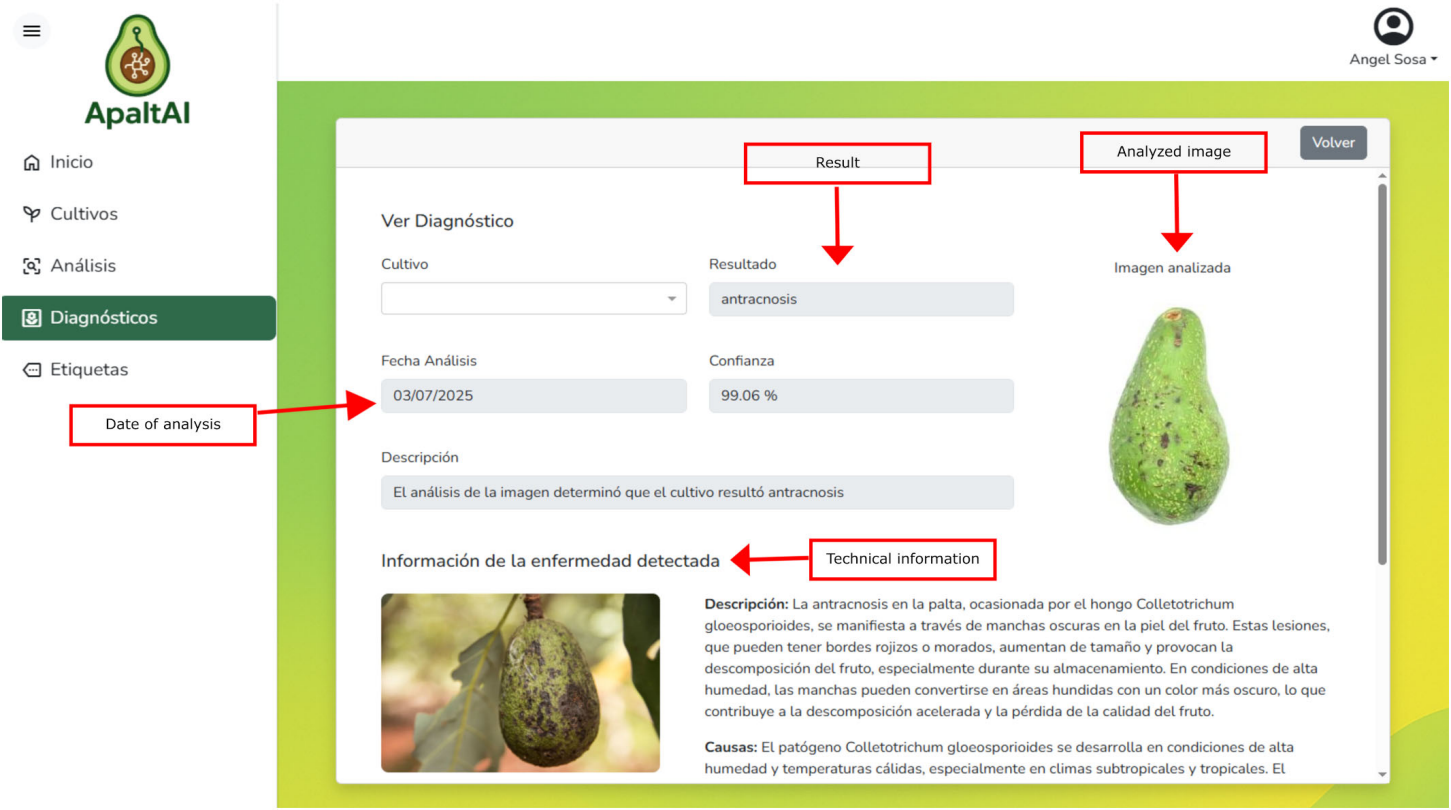
Interface of the diagnosis module.

- *Diagnosis module* is designed to deliver actionable and trustworthy diagnostic information to support agricultural decision-making (see **Figure 6**). For each analysis, the interface presents the primary diagnosis (e.g., ‘Healthy’, ‘Anthracnose’, ‘Scab’) alongside a model confidence score (derived from VotingBS scheme), providing users with a transparent measure of the system’s certainty. To enhance interpretability, each result is accompanied by detailed technical information on the identified disease —including characteristic symptoms, causal agents, and conditions favoring its development— along with science-based agronomic management recommendations. Furthermore, the module maintains a complete chronological history of all diagnoses for a given crop, enabling users to track disease progression and treatment efficacy over time. This combination of a quantifiable confidence metric, explanatory agronomic context, and historical tracking is explicitly designed to bridge the gap between algorithmic output and informed field decision, thereby fostering user trust and interpretability.

### 3.4 Validation strategy

A detailed framework was followed during the validation to ensure reliable findings. This process included four main stages: (1) dataset description, (2) definition of evaluation metrics, (3) execution of controlled experiments across different models, and (4) comparative analysis of the results. Each stage was carefully documented and adapted to address specific challenges in avocado disease detection, thereby ensuring both technical and agricultural relevance.

#### 3.4.1 Dataset

**Figure 7:**
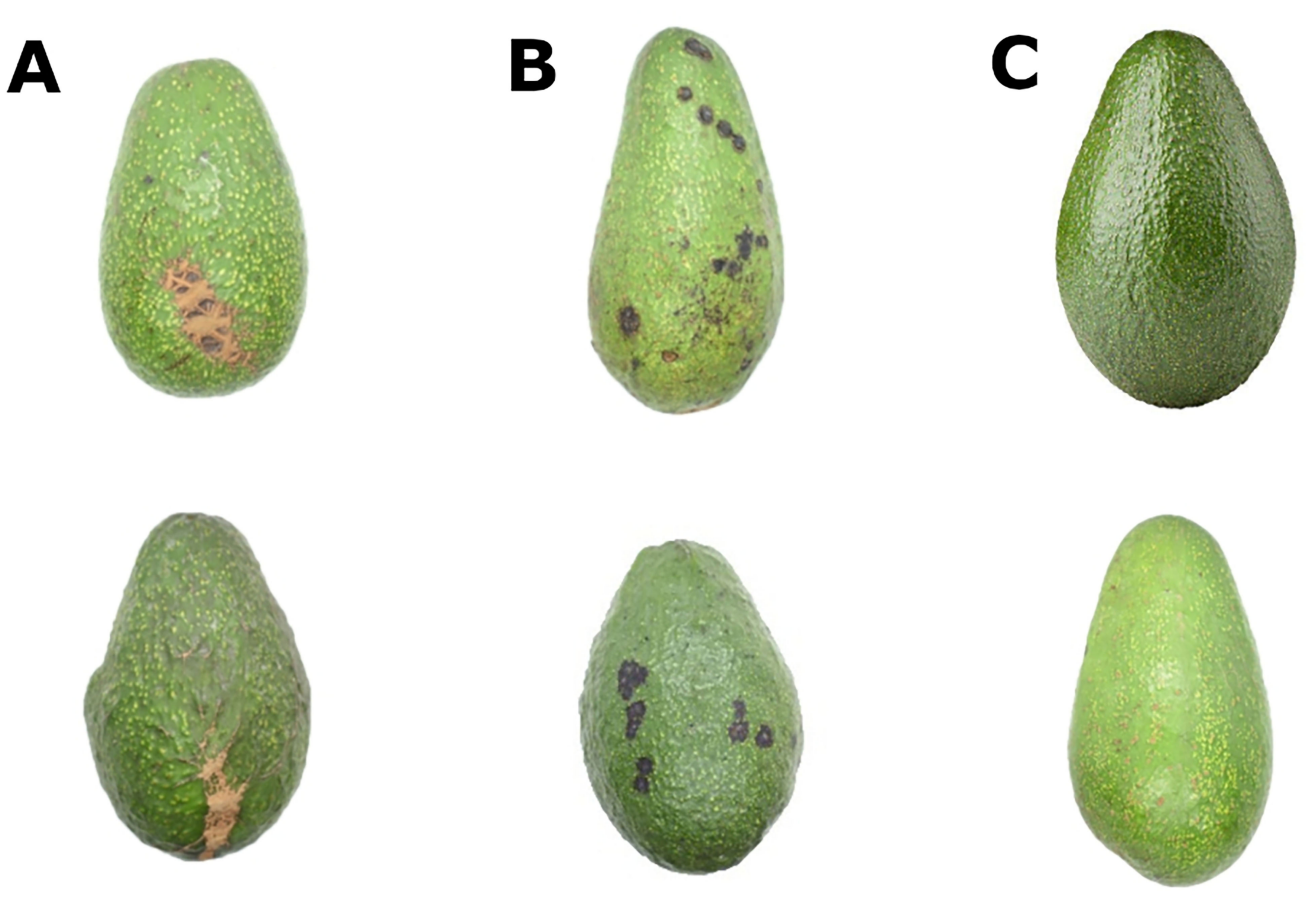
Avocado fruit images by class: **(A)** Scab, **(B)** Anthracnose, **(C)** Healthy.

This research utilized a dataset of avocado fruit images compiled from multiple sources: 351 images from the public dataset available on Kaggle (camposfe1/clasificacion-de-enfermedades-con-deep-learning), 63 images from the “Hass” Avocado Ripening Photographic Dataset (Xavier et al., 2024), supplemented by 260 additional images to achieve equitable distribution among the three classification groups: healthy, affected by scab, and affected by anthracnose. These additional images were sourced from various online platforms and verified by agronomy specialists. **Figure 7** shows some examples of these images.

The dataset was subjected to the following preprocessing pipeline. RGB input images were resized to the required input dimensions for each architecture: 224×224 pixels for ResNet50, VGG16, and DenseNet121; 299×299 for InceptionV3; and 260×260 for EfficientNetB2. Subsequently, pixel values were normalized using the specific preprocessing methods provided by each model. Data augmentation was implemented during training using only photometric transformations that conserve crucial diagnostic features, including brightness adjustments of ±0.2 and contrast adjustments of ±0.5. This conservative strategy was chosen deliberately. The key diagnostic features for anthracnose and scab—such as lesion color, texture, and precise morphological boundaries—are sensitive to geometric distortions (e.g., aggressive cropping or rotation) which could alter their relative scale or orientation, potentially confusing the model. The selected thresholds for brightness (± 0.2) and contrast (± 0.5) were empirically set to simulate realistic variations in natural lighting and camera capture conditions without causing unrealistic over- or under-exposure that would distort color-based diagnostic cues. While more extensive augmentation strategies (including geometric transformations) are valuable for enhancing robustness to viewpoint changes, they were reserved for future work with larger, more diverse field datasets where such variability is inherent. The preprocessing steps maintained the original dataset size, with the total number of processed images kept constant at 674. Photometric variations were generated in real-time for each image during the different training epochs, without creating additional physical copies. **Table 3** summarizes the dataset characteristics before and after preprocessing.

**Table 3:** Characteristics of the original and preprocessed dataset.

Characteristic	Original	Preprocessed
Dimension	Variable	224 x 224 260 x 260 299 x 299
Pixel range	0 - 255	0 - 1
Total images	674	674
Healthy images	282	282
Scab images	196	196
Anthracnose images	196	196

The preprocessed dataset was divided into two subsets with the following distribution: 85% for training and 15% for validation. A hold-out validation strategy was employed instead of k-fold cross-validation due to the substantial computational cost associated with training and fine-tuning five distinct deep learning architectures multiple times. This approach provides a clear and computationally efficient partition for unbiased performance evaluation and model selection, which is a standard practice for the comparative analysis of deep learning architectures in similar studies. The assembled dataset of 674 images provides a foundational basis for the comparative development and validation of the proposed VotingBS architecture against established model benchmarks. While larger datasets exist for other crops, this collection is of comparable size to foundational works in specialized agricultural vision tasks (e.g., Turkoglu et al., 2022: 1,192 images; Hari and Singh, 2023: 1,791 images) and is sufficient for the primary objective of this study: to demonstrate the efficacy and comparative advantage of a novel decision architecture under controlled experimental conditions. The limitations of this dataset regarding generalization to uncontrolled field environments are explicitly addressed in the Discussion (Section 5).

#### 3.4.2 Evaluation metrics

To evaluate the model’s performance, standard image classification metrics were employed, including precision, recall, and accuracy, as used in various agricultural studies (Al-Wesabi et al., 2022; Polly and Devi, 2024; Moussafir et al., 2022). These metrics are defined and formulated as follows:

(4) $$Precision\;=\;\frac{TP}{TP\;+\;FP}$$

(5) $$Recall\;=\frac{TP}{TP\;+\;FN}$$

(6) $$Accuracy=\frac{TP\;+\;TN}{TP\;+\;TN\;+\;FP\;+\;FN}$$

Where, precision (**Equation 4**) measures how well the model correctly classifies an avocado into each category (scab, anthracnose, or healthy), minimizing confusion between classes; recall metric (**Equation 5**) quantifies how well the model can find all positive cases for every class of avocado, ensuring that no instances of scab or anthracnose are missed; accuracy (**Equation 6**) reflects the overall percentage of avocado images correctly classified (scab, anthracnose, or healthy) by the model.

#### 3.4.3 Experiments

Five CNN models —ResNet50, InceptionV3, EfficientNetB2, VGG16, and DenseNet121— were evaluated, with hyperparameters optimized through literature review and empirical testing (**Table 4**). To mitigate the risk of overfitting given the dataset size, two strategies were employed: (1) Transfer learning using ImageNet pre-trained weights, which provides models with robust generic feature extractors from the start, and (2) Photometric data augmentation (brightness and contrast adjustments) during training, which introduces variability and improves model invariance to lighting conditions. These strategies were chosen to enhance generalization within the constraints of the available data, allowing for a robust comparative evaluation of the proposed architectures.

**Table 4:** Hyperparameters of the singular DL models.

ResNet50	InceptionV3	EfficientNetB2	VGG16	DenseNet121
Batch size = 32 weights = Imagenet Input shape = 224 x 224 include_top = False Dense activation = relu Learning rate = 0.001 Optimizer = SGD Epochs = 80	Batch size = 32 weights = Imagenet Input shape = 299 x 299 include_top = False Dense activation = relu Learning rate = 0.001 Optimizer = SGD Epochs = 80	Batch size = 32 weights = Imagenet Input shape = 260 x 260 include_top = False Dense activation = relu Learning rate = 0.001 Optimizer = SGD Epochs 80	Batch size = 32 weights = Imagenet Input shape = 224 x 224 include_top = False Dense activation = relu Learning rate = 0.001 Optimizer = SGD Epochs = 80	Batch size = 32 weights = Imagenet Input shape = 224 x 224 include_top = False Dense activation = relu Learning rate = 0.001 Optimizer = SGD Epochs = 80

The five CNN models were implemented in a development environment equipped with an AMD Ryzen 7 5700X eight-core CPU, 16 GB RAM, and 1TB SSD storage, using Python. Experiments were executed under this hardware configuration, and the hyperparameters of each architecture were manually tuned based on performance across the validation subset. Three scenarios were considered in the model’s evaluation:

- *Singular*: Classification using the five individual multiclass models.
- *Hybrid*: Classification using the hybrid multiclass models VGG16+RF and DenseNet121+RF, which demonstrated superior performance compared to other hybrid combinations of two individual models.
- *Voting*: Classification using a voting scheme (involving all five singular models) and the VotingBS model.

Following the structure of the VotingBS model, each of the 5 models was trained in two sequential phases. A binary classification task was performed in the first phase, where images were categorized as either healthy or unhealthy. In the second phase, the same architecture was reused to classify between the two main diseases under study: scab and anthracnose.

## 4 Results

**Figure 8:**
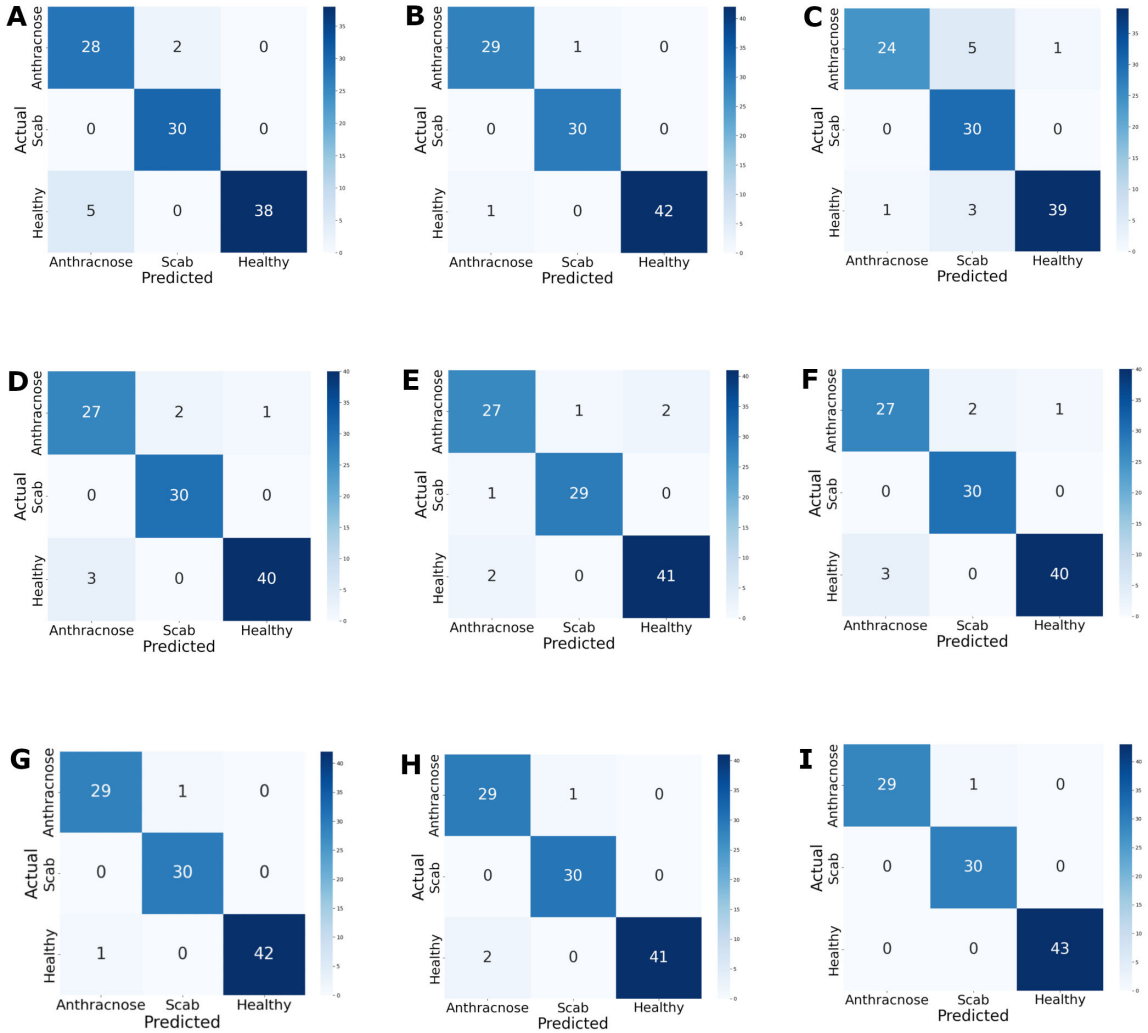
Confusion matrices of the evaluated classification architectures: **(A)** ResNet50, **(B)** VGG16, **(C)** InceptionV3, **(D)** EfficientNetB2, **(E)** DenseNet121, **(F)** DenseNet121 + RF, **(G)** VGG16 + RF, **(H)** Voting, **(I)**. VotingBS.

**Figure 8** presents the confusion matrices of the nine DL models implemented for the three avocado disease categories. Among them, five are singular models, three are hybrid models —one of which is a multiclass voting model— and the last is the proposed binary sequential voting architecture.

The accuracy and loss curves across training epochs for the five singular models, during both training and validation, are provided in **Table 5**. The training loss function stabilizes around epoch 50 for all models, except for ResNet50, which stabilizes earlier around epoch 10. However, during validation, EfficientNetB2 shows better loss stabilization. Additionally, it is noted that validation accuracy remains lower than training accuracy across all models, with VGG16 demonstrating the most consistent convergence.

**Table 5:** Accuracy and loss per epoch for singular DL models.

DL Model	Accuracy	Loss
ResNet50	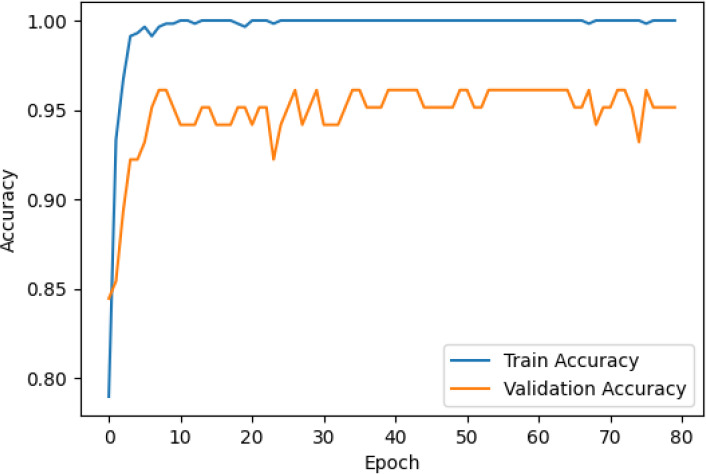	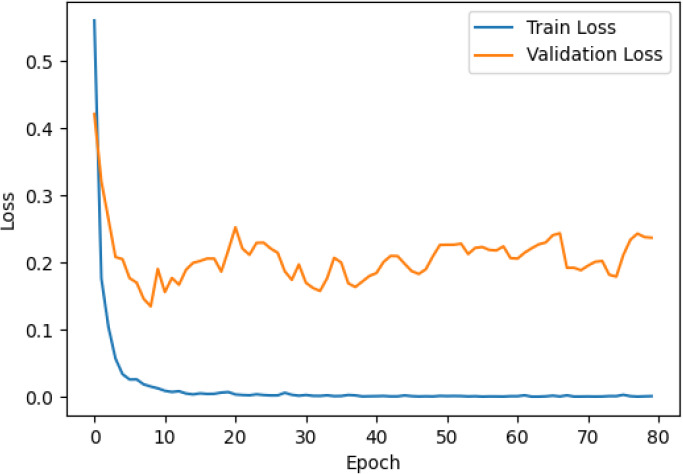
VGG16	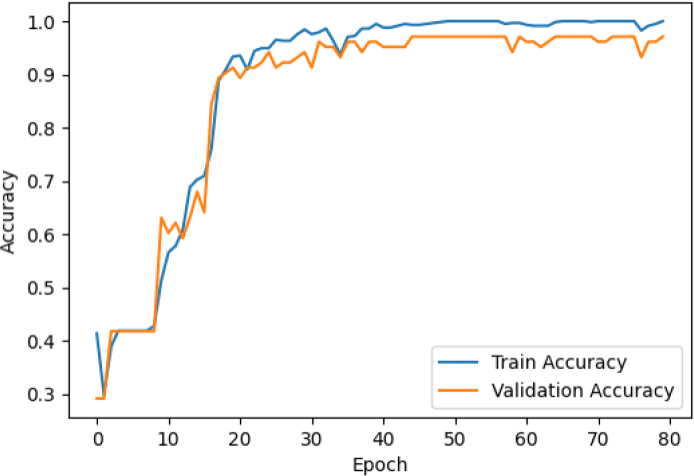	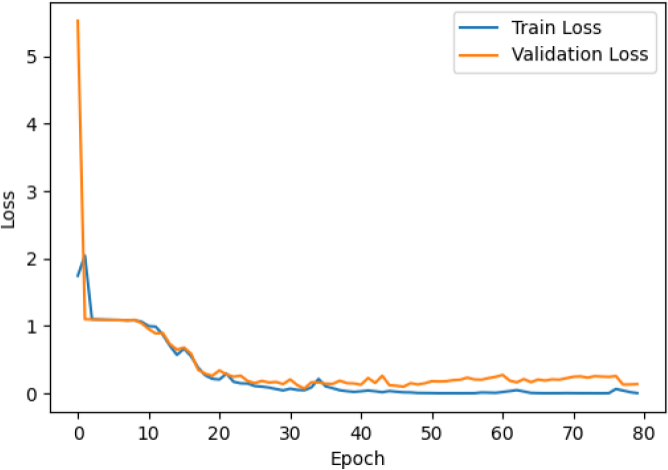
InceptionV3	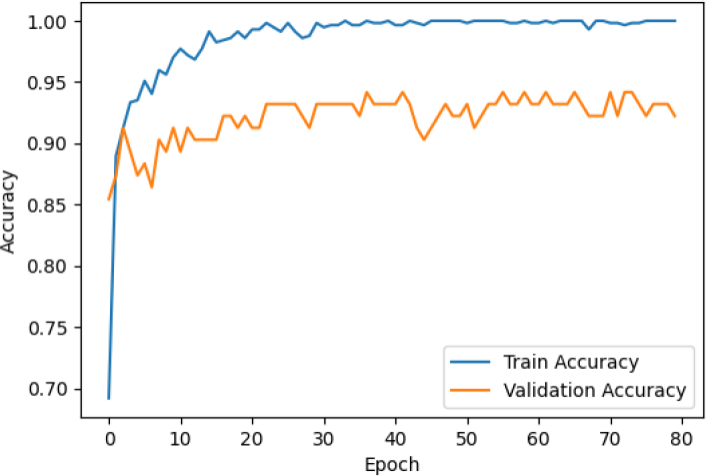	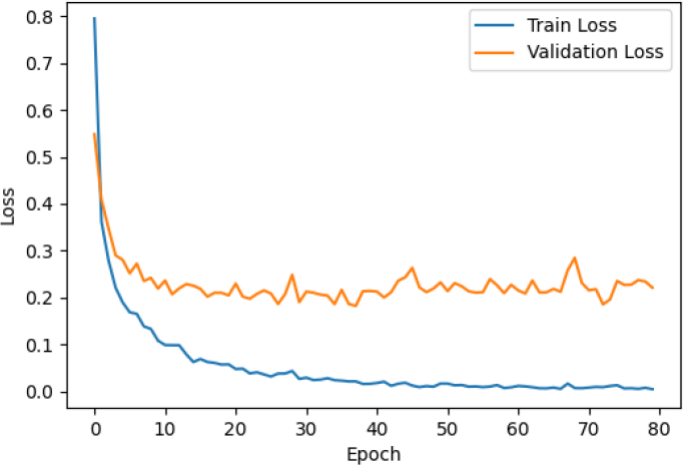
EfficientNetB2	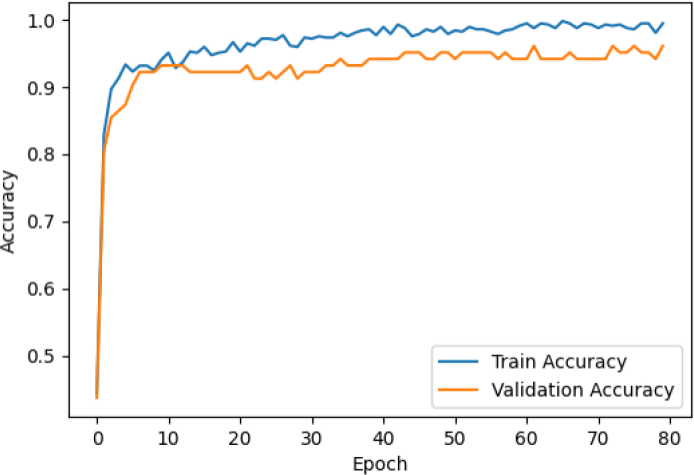	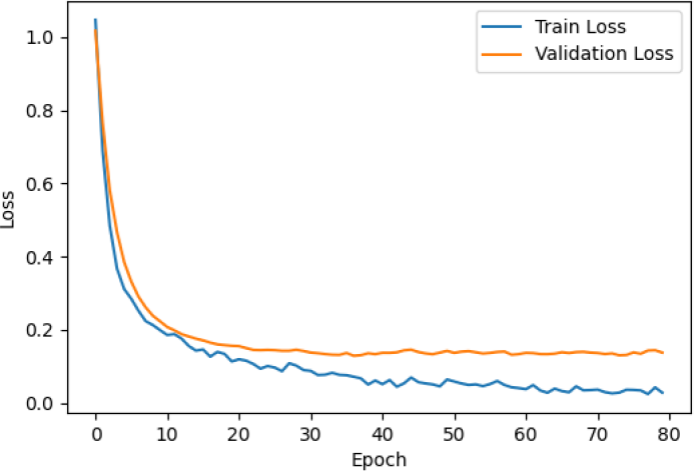
DenseNet121	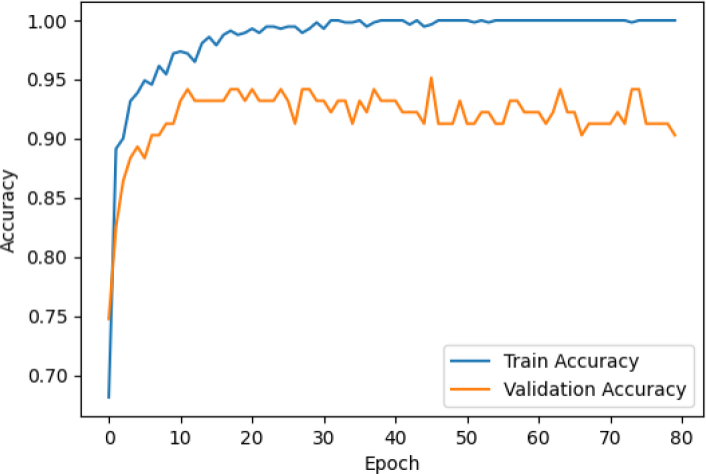	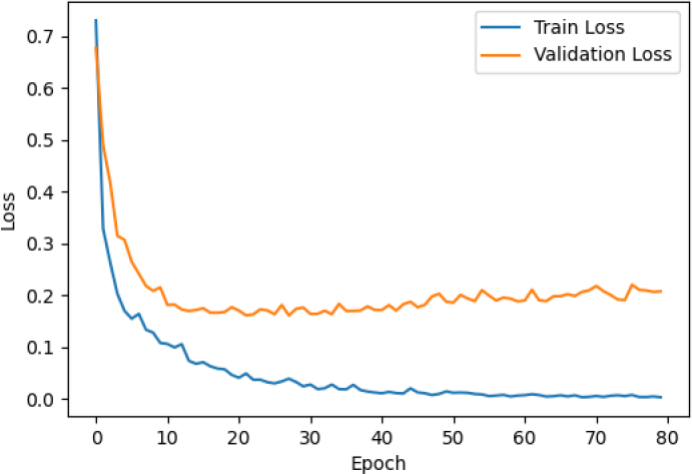

The performance metrics of the DL models under the three evaluation scenarios are shown in **Table 6**. In the singular scenario, VGG16 achieved the best results, with 97.18% precision, 97.09% recall, and 97.09% accuracy. In the hybrid model scenario, the VGG16 + RF combination yielded superior performance, achieving 97.81% precision, 98.11% recall, and 98.06% accuracy. The findings also demonstrate that the proposed VotingBS consistently outperformed both the individual pre-trained architectures and the hybrid models across all metrics, reaching 98.92% precision, 98.89% recall, and 99.03% accuracy. To provide a foundational baseline and contextualize the advancement of our proposed models, **Table 6** also includes the performance of standard machine learning methods (CNN, RF, SVM, MLP) as reported by Campos-Ferreira and González-Camacho, 2021, 2023) on a subset of the same dataset used in this study.

**Table 6:** Comparative performance of models across different architectural scenarios.

Scenarios	Method	Precisión	Recall	Accuracy
Singular	CNN (Campos-Ferreira and González-Camacho, 2021)	79.33%	85.00%	87.00%
	RF (Campos-Ferreira et al., 2023)*	98.00%	97.67%	98.00%
	SVM (Campos-Ferreira et al., 2023)*	97.67%	97.00%	97.00%
	MLP (Campos-Ferreira et al., 2023)*	98.03%	98.00%	98.00%
	ResNet50	95.53 %	95.15 %	95.15 %
	VGG16	97.18 %	97.09 %	97.09 %
	InceptionV3	92.66 %	92.23 %	92.23 %
	EfficientNetB2	96.33 %	96.12 %	96.12 %
	DenseNet121	90.91 %	90.29 %	90.29 %
Hybrid	VGG16 + RF	97.81 %	98.11 %	98.06 %
	DenseNet121 + RF	94.25 %	94.17 %	94.17 %
Voting	Voting	97.18 %	97.09 %	97.09 %
	VotingBS	98.92 %	98.89 %	99.03 %

*Uses a subset of the dataset employed in this study.

## 5 Discussion

This research proposes a smart detection system for avocado fruit diseases using image analysis. The system is based on hybrid DL models and consists of three main modules (crops, analysis, and diagnosis) and is designed to facilitate intuitive disease identification and crop monitoring. The cultivation module enables users to register notes and create new crop entries, while the image preprocessing and disease identification of the fruit are handled by the analysis module. The diagnosis module provides access to the current and historical health status of each fruit, including diagnosis date, identified disease, causal agents, and recommended treatments.

This study introduces the VotingBS architecture, an innovative two-phase sequential voting scheme designed to optimize disease diagnosis in avocado crops. In the first phase, five DL models (ResNet50, VGG16, InceptionV3, EfficientNetB2, and DenseNet121) perform a binary classification (healthy vs. diseased fruit), while in the second phase, they specifically discriminate between anthracnose and scab. Experiments were performed using a collection of 674 images (571 for training and 103 for validation). The results demonstrated the superiority of this method: while the best singular multiclass model (VGG16) achieved 97.18% precision and 97.09% recall and accuracy, and its hybrid version with Random Forest (VGG16+RF) improved these results by +0.6%, the VotingBS architecture significantly outperformed all alternatives, reaching 98.92% precision, 98.89% recall, and 99.03% accuracy, thereby surpassing the best results reported in the literature.

The superiority of the VotingBS approach lies in its sequential architecture, which decomposes the diagnostic task into two clearly defined stages, thereby reducing cumulative errors typically observed in conventional models. This strategy not only proved effective in the presented case study but also establishes a promising paradigm for its application in other crops affected by multiple pathologies. The results suggest that breaking down complex tasks into simpler subproblems —combined with weighted voting schemes— can offer significant advantages in diagnostic accuracy over traditional approaches.

### 5.1 Limitations and future work

The performance of the VotingBS architecture, while superior in our experiments, must be interpreted within the constraints of the dataset used. The primary limitation is the dataset’s size (n=674) and composition, which originates from mixed sources and does not include cases of disease co-infection or very early symptoms. Consequently, the reported high accuracy reflects optimal performance on a curated dataset and serves as proof-of-concept. Therefore, the primary direction for future work is the validation of the model’s generalization capability on a larger, prospectively collected field image corpus that captures the full heterogeneity of real orchards, including diverse lighting, occlusions, and complex disease presentations. In parallel, the next critical phase for the ApaltAI system is its operational validation, encompassing formal performance evaluation under high-load and poor-connectivity conditions, as well as extensive User Acceptance Testing (UAT) with avocado farmers to ensure its practical usability and adaptability in the field. These steps are essential to transition the integrated system from a robust prototype to a reliable agricultural tool.

## 6 Conclusions

This research developed an innovative system for disease detection in avocado crops, combining a hybrid binary sequential ensemble architecture (VotingBS) with a supportive web application. The core innovation lies in its two-stage decision architecture: initially classifying fruits as healthy or unhealthy through the voting of five deep learning models and subsequently identifying the specific disease (anthracnose or scab) through a second weighted voting process among other five specialized models. This hierarchical approach demonstrated outstanding performance —98.92% precision, 98.89% recall, and 99.03% accuracy— significantly surpassing both singular and hybrid models documented in previous studies.

Although the results highlight the system’s potential, its current scope is constrained by the limitations of the dataset used. Nevertheless, this work lays the groundwork for key future developments: (1) integration with precision agriculture systems to enable parcel-level monitoring, and (2) scaling VotingBS to include additional and co-occurring diseases. These advancements would position the proposed application as an intelligent, comprehensive, and scalable solution for the sustainable phytosanitary management of avocado crops.

## Data Availability

The original contributions presented in the study are included in the article/supplementary material. Further inquiries can be directed to the corresponding author.
